# A plant-infecting subviral RNA associated with poleroviruses produces a subgenomic RNA which resists exonuclease XRN1 *in vitro*

**DOI:** 10.1016/j.virol.2021.11.002

**Published:** 2021-11-17

**Authors:** A.J. Campbell, John R. Anderson, Jeffrey Wilusz

**Affiliations:** a Department of Microbiology and Molecular Genetics, University of California, Davis, CA, 95616, USA; b Department of Microbiology, Immunology and Pathology, Colorado State University, Fort Collins, CO, 80523, USA

**Keywords:** Tombusvirus, XRN1, Coat-dependent RNA replicon, Subviral RNA, tlaRNA, Subgenomic RNA

## Abstract

Subviral agents are nucleic acids which lack the features for classification as a virus. Tombusvirus-like associated RNAs (tlaRNAs) are subviral positive-sense, single-stranded RNAs that replicate autonomously, yet depend on a coinfecting virus for encapsidation and transmission. TlaRNAs produce abundant subgenomic RNA (sgRNA) upon infection. Here, we investigate how the well-studied tlaRNA, ST9, produces sgRNA and its function. We found ST9 is a noncoding RNA, due to its lack of protein coding capacity. We used resistance assays with eukaryotic Exoribonuclease-1 (XRN1) to investigate sgRNA production via incomplete degradation of genomic RNA. The ST9 3’ untranslated region stalled XRN1 very near the 5’ sgRNA end. Thus, the XRN family of enzymes drives sgRNA accumulation in ST9-infected tissue by incomplete degradation of ST9 RNA. This work suggests tlaRNAs are not just parasites of viruses with compatible capsids, but also mutually beneficial partners that influence host cell RNA biology.

## 1. Introduction

Tombusvirus-like associated RNAs (tlaRNAs) are autonomously replicating + ssRNAs of about 2.8 kb that are most closely related to viruses of the family *Tombusviridae* (Campbell et al., 2020). Encoding only an RNA dependent RNA polymerase (RdRp) which enables their independent replication, tlaRNAs rely on one or more co-infecting viruses, typically poleroviruses, for systemic movement and for aphid transmission. TlaRNAs gain these functions by utilizing the coat proteins produced by the co-infecting virus for their own encapsidation (Chin et al., 1993; Passmore et al., 1993). In coinfections with umbraviruses, tlaRNAs can gain systemic movement, but cannot be transmitted by aphids without a coinfecting polerovirus to supply coat proteins to the mixed infection (Passmore et al., 1993; Taliansky et al., 2003). TlaRNAs produce an abundant subgenomic RNA (sgRNA) of roughly 470 nucleotides that accumulates as both positive and negative sense RNA during infection (Campbell et al., 2020). The function of this sgRNA in infection and the mechanism underlying its production remain to be elucidated.

Subgenomic RNAs have been shown to play various critical roles in viral infections, from encoding proteins as messenger sgRNAs, fulfilling regulatory functions in replication and transcription, and aiding in suppression of host immune responses (Sztuba-Solińska et al., 2011; Shen and Miller, 2004; Pompon et al., 2017). Discovering the function of virally produced sgRNAs is key to a holistic understanding of the infection process of a virus, and in understanding how one virus or subviral agent may interact with another during co-infections. Viral satellites and subviral entities have been noted to exert an influence on the ecological and evolutionary dynamics of the viruses they associate with, with effects ranging from synergism to antagonism (Moreno and López-Moya, 2020). In the case of tlaRNAs, including the most well-studied, designated ST9, increases in symptom severity have been observed in co-infections with poleroviruses in some host plants (Peng et al., 2021; Sanger et al., 1994; Falk and Duffus, 1984). Interestingly, increased symptom severity upon co-infection of tlaRNAs and poleroviruses has been observed to be host-dependent (unpublished observations).

Accumulation of a large amount of the sgRNA produced by ST9 and other tlaRNAs during infections underscores the potential importance of this sgRNA and led to investigations into their potential as a messenger sgRNA or its function as a noncoding RNA. Synthesis of sgRNAs has been extensively studied in plant systems, and different mechanisms for their generation have been proposed (Sztuba-Solińska et al., 2011). Two confirmed methods for sgRNA production in plant viruses are internal initiation of transcription on the negative strand and premature termination during transcription of the negative strand (Miller and White, 2006). A third method for sgRNA production, described most extensively for production of noncoding sgRNAs, is incomplete degradation of the viral genomic RNA by a host exonuclease (Tycowski et al., 2015).

During internal initiation of transcription, the viral RdRp binds to a non-terminal nucleotide sequence on the negative (non-genome) RNA strand and begins transcription at this internal locus, thus generating subgenomic RNAs. A key feature underlying this mechanism for sgRNA production is a subgenomic promoter, the nucleotide sequence that is recognized and bound by the RdRp for initiation (Miller and Koev, 2000; Siegel et al., 1997). The promoter can be located either upstream or downstream of the sgRNA 5’ end, although most known subgenomic promoters map to a region just upstream of the 5’ end of the sgRNA and may proceed a handful of nucleotides downstream into the sgRNA sequence itself (Levis et al., 1990; Van der Vossen et al., 1995; Johnston and Rochon, 1995). Premature termination can occur during transcription of negative strand RNA by the viral RdRp, resulting in negative sense sgRNAs with 5’ ends coterminal with the genomic 3’ end. These RNAs can then be used as templates for transcription into positive sense sgRNAs and can serve as subgenomic mRNAs (Sztuba-Solińska et al., 2011; White, 2002; Choi and Andrew White, 2002).

Incomplete degradation of viral genomes by host nucleases can lead to sgRNA production. Many + ssRNA viruses replicate in the cytoplasm and lack features such as 5’ caps and poly(A) tails which shield RNAs from endogenous decay, thus these viruses must find ways to evade the host RNA decay machinery (Dickson and Wilusz, 2011). The primary exonuclease responsible for RNA decay in the cytoplasm is XRN1 (in plants, XRN4 is the homolog of XRN1), which requires its substrates to possess a monophosphate at the 5’ end in order to initiate decay, and proceeds in a 5’ to 3’ direction, reducing its substrates to mononucleotides (Slonchak and Khromykh, 2018). XRN1’s processing activity is powerful; it has been shown to degrade highly structured RNAs such as internal ribosome entry sites (IRES) and ribosomal RNA (Pijlman et al., 2008). Viral resistance to XRN1 has been studied most thoroughly for members of the genus *Flavivirus,* and the crystal structure of the RNA elements responsible for stalling XRN1 was determined for Murray Valley encephalitis virus and Zika virus (Chapman et al., 2014; Akiyama et al., 2016). RNA secondary and tertiary structures generate a conformation which physically blocks and stalls the enzyme (Chapman et al., 2014; Kieft et al., 2015; Steckelberg et al., 2018a). Multiple viral families have been found to contain elements capable of stalling the enzyme (Steckelberg et al., 2018b; Charley et al., 2018), suggesting that many viruses share the strategy of stalling XRN1 as an inherent part of their biology. Recently, the umbravirus *Opiumpoppy mosaic virus* (OPMV) was found to generate a translated sgRNA via XRN’s exonucleolytic activity (Ilyas et al., 2021). In addition to stalling XRN1, many of the small RNAs produced by the enzyme’s decay activity have been shown to play important roles in defense against the host immune system, thereby creating a more favorable environment for viral infection (Pompon et al., 2017; Schuessler et al., 2012).

As members of the family *Tombusviridae,* tlaRNAs fall into the group of +ssRNAs especially susceptible to host RNA decay since they possess neither 5’ caps nor poly(A) tails (Miller et al., 2002). Recent work has demonstrated a number of plant-infecting RNA viruses possess structured RNA elements which resist degradation by XRN1 and thus lead to accumulation of decay intermediates during infection (Steckelberg et al., 2018b; Gunawardene et al., 2019; Dilweg et al., 2019). In the current work, the mechanism of sgRNA generation of the tlaRNA ST9 was examined using mutational analyses and *in vitro* XRN1 resistance assays. The function of the sgRNA was investigated using mutational analysis to determine whether a protein might be produced from the sgRNA. The results of our study provide strong evidence that the ST9 sgRNA is a noncoding RNA not produced via the action of a subgenomic promoter but through incomplete degradation of the genomic RNA by XRN1.

## 2. Materials and methods

### 2.1. Plant infections and RNA extractions

To assess both wild type ST9 and generated mutants’ dynamics *in planta,* the *Agrobacterium tumefaciens* (now known as *Rhizobium radiobacter,* but referred to in this report as *A. tumefaciens*) strain GV3101 was used to infect plants with an infectious clone of ST9 designated JL89:ST9 and JL89:ST9-derived mutants (Campbell et al., 2020). Bacteria were transformed, cultured, and resuspended to an A_600_ of 0.8 (Lindbo, 2007). Bacterial suspensions were syringe infiltrated into transgenic *Nicotiana benthamiana* (*N. benthamiana*) plants constitutively expressing the turnip mosaic virus silencing suppressor protein HC-Pro to increase viral RNA and protein accumulation, as done previously (Wang et al., 2009; Qiao and Falk, 2018; Qiao et al., 2018). 0.1g of infiltrated tissue was harvested 4 days post inoculation (dpi) and flash frozen with liquid nitrogen. RNA was extracted with TRIzol as per manufacturer’s protocol (Thermo Fisher Scientific, Waltham MA).

### 2.2. Northern blot hybridizations

Northern blotting was performed to assess RNA production and accumulation. Creation of radioactive probes and northern blotting procedures were done as published previously (Matsumura et al., 2019). All hybridizations were performed at 65°C overnight, with a probe concentration of 2 × 10^6^cpm/ml. Primers used in probe creation are listed in Supplementary Table 1.

### 2.3. Mutational analyses

Mutants were created to investigate involvement of a potential subgenomic promoter in sgRNA production, as well as to examine the protein coding capacity of the P4 ORF. Constructs were created using mutagenic primers listed in Supplementary Table 1 using the NEBuilder HiFi Cloning method with the JL89:ST9 infectious clone as the template (New England Biolabs, Ipswich, MA). Agroinfiltrations and RNA extractions were performed as above, and tissue was harvested from infiltrated areas at 4 dpi. RNA extracts were analyzed by northern hybridization as described above. To ensure that mutations were maintained throughout the duration of the experiments, aliquots of the RNA used in the northern hybridizations were treated with RNAse-free DNAse I (Qiagen) and column-cleaned with the Zymo RNA Clean and Concentrator kit (Zymo). Treated RNAs were normalized to the same concentration and cDNAs were created using the High Capacity RT reverse transcriptase kit (Applied Biosystems, Waltham, MA). cDNAs and mock cDNA preparations incubated without reverse transcriptase were submitted to PCR reactions with primers flanking the mutation/deletion sites. Products were both analyzed in 1% agarose gels and sent for sequencing.

### 2.4. XRN1 in vitro decay assays

To test whether ST9 could resist XRN1 degradation, a region of ST9 sequence (nucleotides 2151–2563) beginning just upstream of the 3’ UTR and spanning to downstream of the sgRNA 5’ end was directly subjected to challenge by the enzyme *in vitro*. The XRN1 *in vitro* decay assays were conducted as published previously (Moon et al., 2012, 2015). Templates used for *in vitro* transcription were either synthesized by Integrated DNA Technologies (IDT, Coralville, Iowa) or have been previously described (Moon et al., 2012). Samples of the decay reactions were collected at the time points indicated, decay products were separated in 5% denaturing PAGE gels containing 7 M urea, and visualized via phosphorimaging as described (Moon et al., 2012). The sequences of the DNA templates for *in vitro* transcription and subsequent decay assays are listed in Supplementary Table 2.

## 3. Results

### 3.1. Characterization of the tlaRNA 3’ UTR and sgRNA

**Fig. 1.**
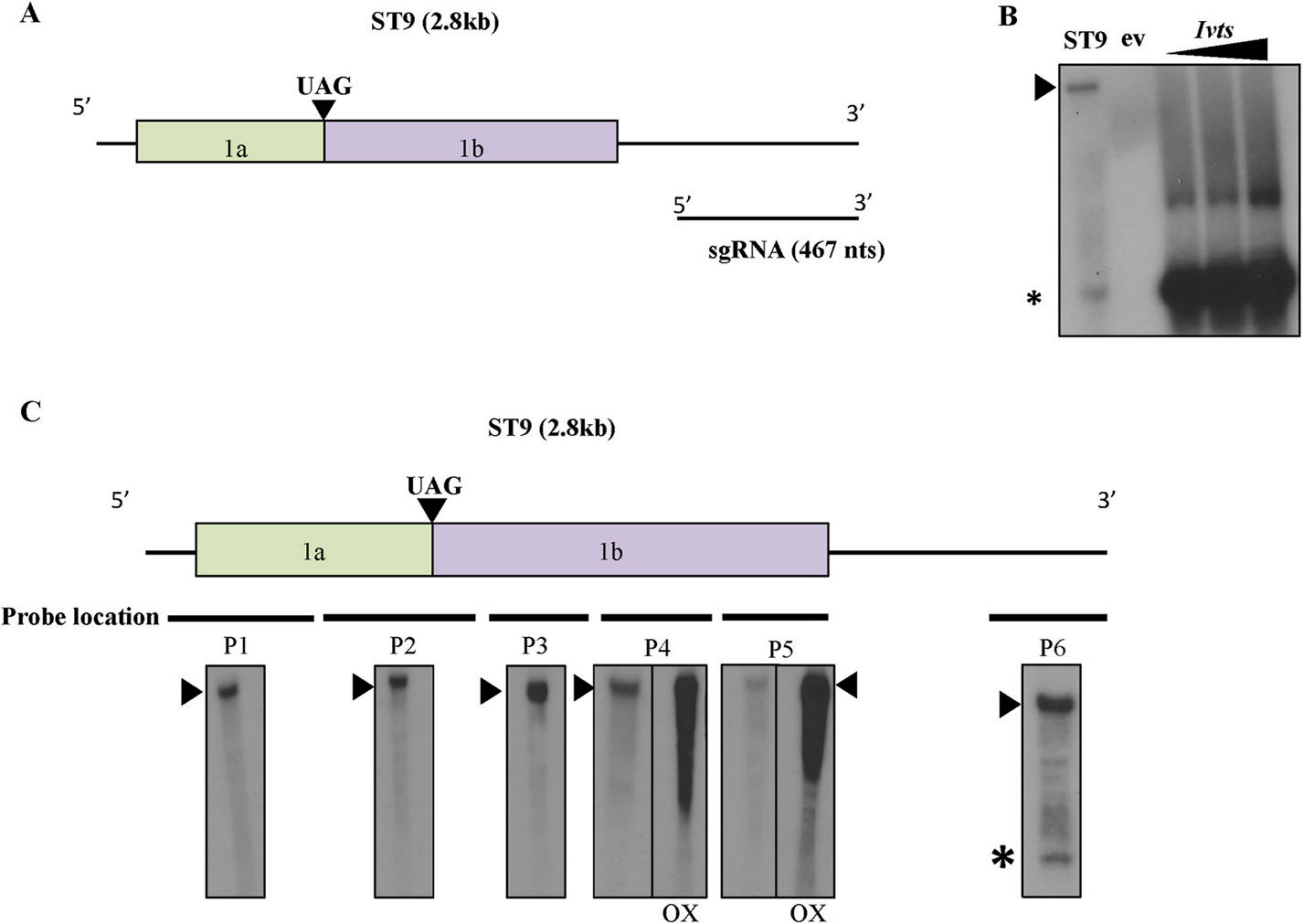
Mapping of the ST9 subgenomic RNA. **A)** Diagram of the tlaRNA ST9 genome. ORFs 1a and 1b are shown in colored boxes; they produce the RdRp via translational readthrough of the UAG amber stop codon, indicated by a filled triangle. The sgRNA is shown beneath its corresponding genome location. **B)** Northern hybridization of ST9 RNA extracted from infected *N. benthamiana,* ST9, empty vector control, ev, and increasing concentrations of *in vitro* transcripts, *Ivts,* corresponding to the 3’ 467 nts of ST9. Transcript concentrations increase left to right 500 ng, 700 ng, 900 ng. The genomic RNA is indicated with a filled triangle, sgRNA is indicated with an asterisk. A positive sense probe was used to detect the negative sense RNA. The upper band seen in the *Ivt* lanes is presumed to be a dimer of the transcript. **C)** The genome of ST9 is shown with thick lines underneath to represent the location of each of the 6 probes used in the northern hybridizations. The exposures of blots using each probe are shown beneath their corresponding lines. Probes 4 and 5 are shown with overexposures, OX, to demonstrate no sgRNA was found using these more 3’ probes. The genomic RNA is indicated with a filled triangle, the sgRNA is indicated with an asterisk.

All studied tlaRNAs have been observed to produce an abundant, similarly sized sgRNA in both positive and negative RNA polarities (Campbell et al., 2020). This sgRNA is highly abundant in the positive sense upon infection, and previous work indicated it is likely to be coterminal with the 3’ end of the genomic RNA (Campbell et al., 2020). There are no predicted open reading frames (ORFs) in tlaRNAs after the one encoding the RdRp, called ORF 1b, thus the remainder of the genome is expected to constitute the 3’ untranslated region (UTR) (Fig. 1A). This is intriguing in that the 3’ UTR is then quite long, comprising roughly 17% of the genome (compare to the 3’ UTR of tomato bushy stunt virus, the type member of the *Tombusviridae,* which comprises only ~ 7% of the genome) (Na et al., 2006). Viral 3’ UTRs are known to play key roles in replication, mRNA stability and translation. In order to elucidate the role that this long 3’ UTR and sgRNA play in tlaRNA infection, we first characterized the sgRNA produced by the ST9 and other tlaRNAs. The 5’ end of the sgRNAs produced by ST9 and three other isolates representing two distinct tlaRNA subclades were determined via 5’ Rapid Amplification of cDNA Ends (RACE). As seen in Table 1, the sgRNAs were shown to be comprised of nearly the entire 3’ UTR and to be ~467–517 nucleotides long.

As a verification that the lower molecular weight RNA species seen in northern hybridizations of ST9 RNA was indeed the sgRNA, RNA transcripts corresponding to the 3’ 467 nucleotides of ST9 were generated *in vitro*. A previously described infectious clone of ST9, termed JL89:ST9 was agroinfiltrated into *Nicotiana benthamiana (N. benthamiana)* plants and infiltrated tissue was harvested four days later and used in hybridizations alongside the *in vitro* transcribed RNA (Campbell et al., 2020). The RNA transcripts and the sgRNA of ST9 migrated to the same position in the gel, confirming the sgRNA size (Fig. 1B). To confirm the genomic location the sgRNA is derived from, six probes of between 340 and 400 nucleotides each were designed to span the length of the ST9 genome. When northern blot hybridizations of ST9 RNA derived from the infected tissue were performed with these probes, only the 3’-most probe was able to hybridize with the sgRNA, while all six probes hybridized with the genomic RNA (Fig. 1C). Therefore we conclude that, in agreement with previous results (Campbell et al., 2020), the ST9 sgRNA is 3’ coterminal with the genomic RNA.

### 3.2. The ST9 sgRNA is likely a noncoding transcript

Examination of the 3’ UTR of ST9 revealed one short ORF of ~3.4 kDa, termed P4. In an effort to explore the function of the ST9 sgRNA and decipher whether it was a messenger sgRNA or served another purpose during infection or coinfection, the P4 ORF was investigated for protein expression. Firstly, the sequence context surrounding the P4 start codon was compared to the modified Kozak context for optimal initiation of translation in plants, which is R(A/C)NaugGC where R is a purine and N is any nucleotide (Hernández et al., 2019; Gupta et al., 2016). The most important positions for translation initiation are −3, the purine, and +4 G. The P4 start codon context is CAUaugGU, which maintains only one of the two critical positions and is thus less than optimal for translation initiation.

**Fig. 2.**
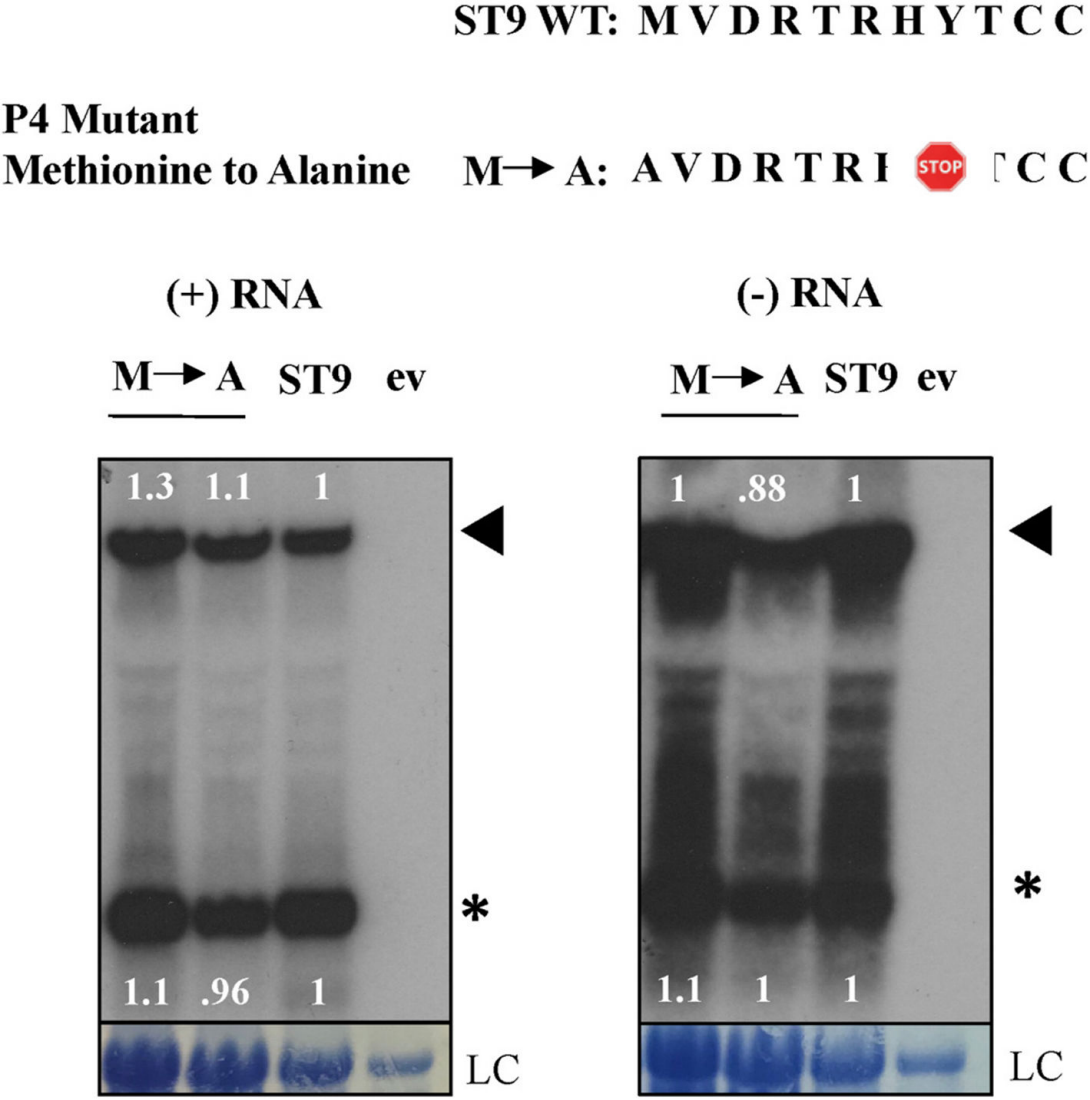
The P4 knock out mutant has no effect on ST9 replication or sgRNA production. The wild type (WT) ST9 P4 translated amino acid sequence is shown above, the amino acid sequence of the mutant is indicated below. The abbreviation for the mutant is listed next to its amino acid sequence. The stop sign symbol indicates an introduced stop codon. Below, northern hybridizations show the mutant in both positive (+) and negative (−) polarities, along with the wild type ST9 and empty vector control, ev. The genomic RNA is indicated with a filled triangle, the sgRNA is indicated with an asterisk. The band intensities were quantified using the ImageJ software package, normalized against their loading controls, and the ratio of each band intensity to the wild type ST9 band intensity is shown above, for the genomic RNA, and below, for the sgRNA. Methylene blue staining of the 18S ribosomal RNA as loading control is shown at bottom, LC.

Next, a mutant was designed to interfere with protein expression from the P4 ORF by eliminating its start codon (Fig. 2). The mutant was inoculated into *N. benthamiana* plants and in agreement with previous observations that tlaRNAs in the absence of a coinfecting virus don’t induce symptoms, infected plants were indistinguishable from wild type. RNA was extracted from infected plants, used in northern hybridizations, and sequencing results confirmed all mutations were maintained throughout the duration of the experiment. The mutant yielded genomic and subgenomic RNA accumulations indistinguishable from wild type ST9. The results of the mutational analysis indicate that a hypothetical protein derived from the P4 ORF has no essential function related to the replication, infection, or sgRNA production of ST9 (Fig. 2).

### 3.3. Subgenomic promoter-driven expression of the ST9 tlaRNA sgRNA is unlikely

**Fig. 3.**
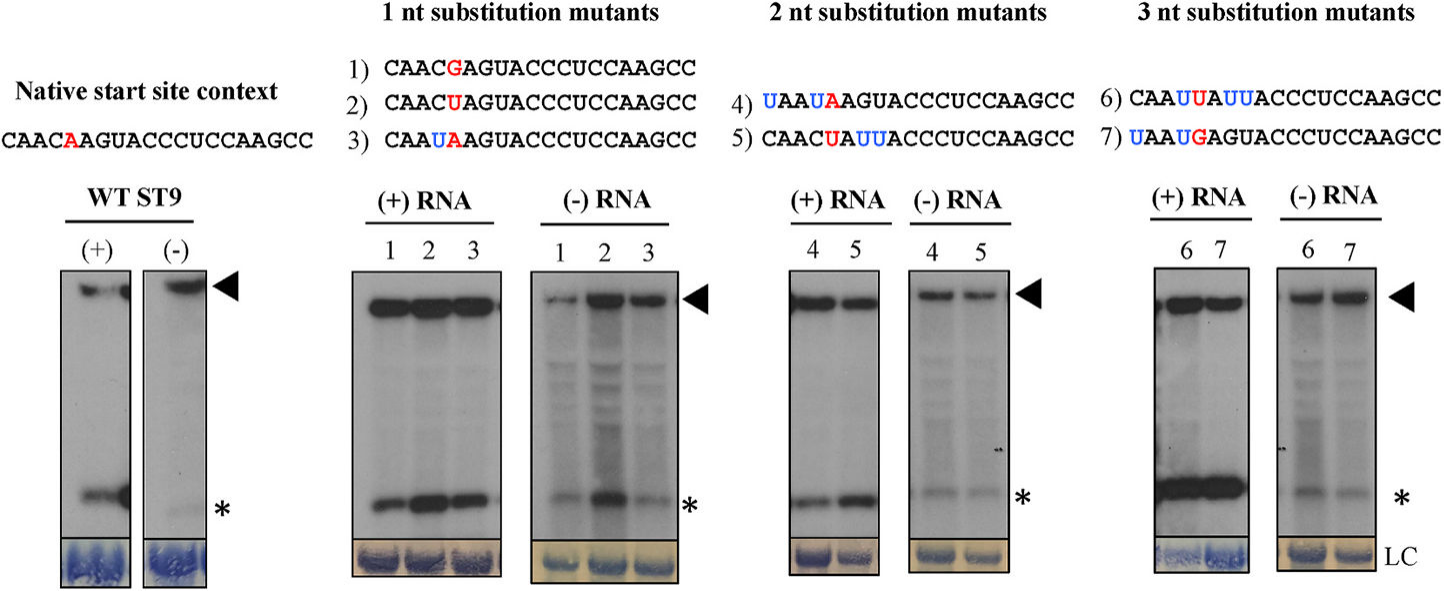
Nucleotide substitution mutations around the 5’ end of the sgRNA do not affect production of the sgRNA. RNA sequences of the 7 substitution mutants are shown above their corresponding northern blots. Mutants are labeled 1–7 as indicated, and the ST9 wild type sequence is shown at the left. The 5’ terminal nucleotide of the sgRNA is indicated in red type. Other substitution mutations are indicated in blue type. Positive (+) and negative (−) sense RNA is shown. Exposures for the (+) RNAs were between 2.5 and 6 h and exposures for the (−) RNAs were between 11 and 16 h. Genomic RNA designated by a filled triangle, sgRNA designated by a asterisk. Methylene blue stained 28S or 18S ribosomal RNA is shown as a loading control (LC) for the positive sense RNAs or negative sense RNAs, respectively.

To investigate internal initiation of transcription as a potential mechanism underlying production of the ST9 sgRNA, experiments targeting the function of a potential subgenomic promoter were undertaken. In many virus species, the promoters responsible for mediating sgRNA generation have been shown to require strict sequence specificity. For example, in TMV, individual substitution mutations in the 5 nucleotides before and after the transcription start site of the I_2_ sgRNA impacted the production of the sgRNA, including abolishing it or reducing its accumulation to around 5% of wild type levels (Grdzelishvili et al., 2000). Thus, mutations in the genome sequence surrounding the 5’ end of the ST9 sgRNA were engineered to disable or alter the function of a potential subgenomic promoter. A series of one, two, and three nucleotide substitution mutants, with the substitutions in the genomic RNA sequence occurring within four nucleotides of the 5’ terminus of the sgRNA were generated (Fig. 3). In some of these the 5’ terminal nucleotide of the sgRNA was altered, and in some mutants, guanine bases were specifically targeted for substitution because previous studies, examining barley yellow dwarf virus and flock house virus respectively, had successfully eliminated sgRNA production employing these methods (Koev and Miller, 2000; Eckerle and Ball, 2002). The constructs were agroinfiltrated into *N. benthamiana* plants and RNA extracted from infiltrated tissue was analyzed via northern blot hybridization for both positive and negative sense RNAs. Sequencing results confirmed all mutations were maintained throughout the duration of the experiment. For all mutants genomic and subgenomic RNA accumulation was indistinguishable from wild type, in both positive and negative RNA polarities (Fig. 3). These results show that the genome sequence immediately surrounding the 5’ end of the sgRNA is tolerant of minor changes and that the identity of the 5’ terminal nucleotide itself is not critical for sgRNA synthesis.

**Fig. 4.**
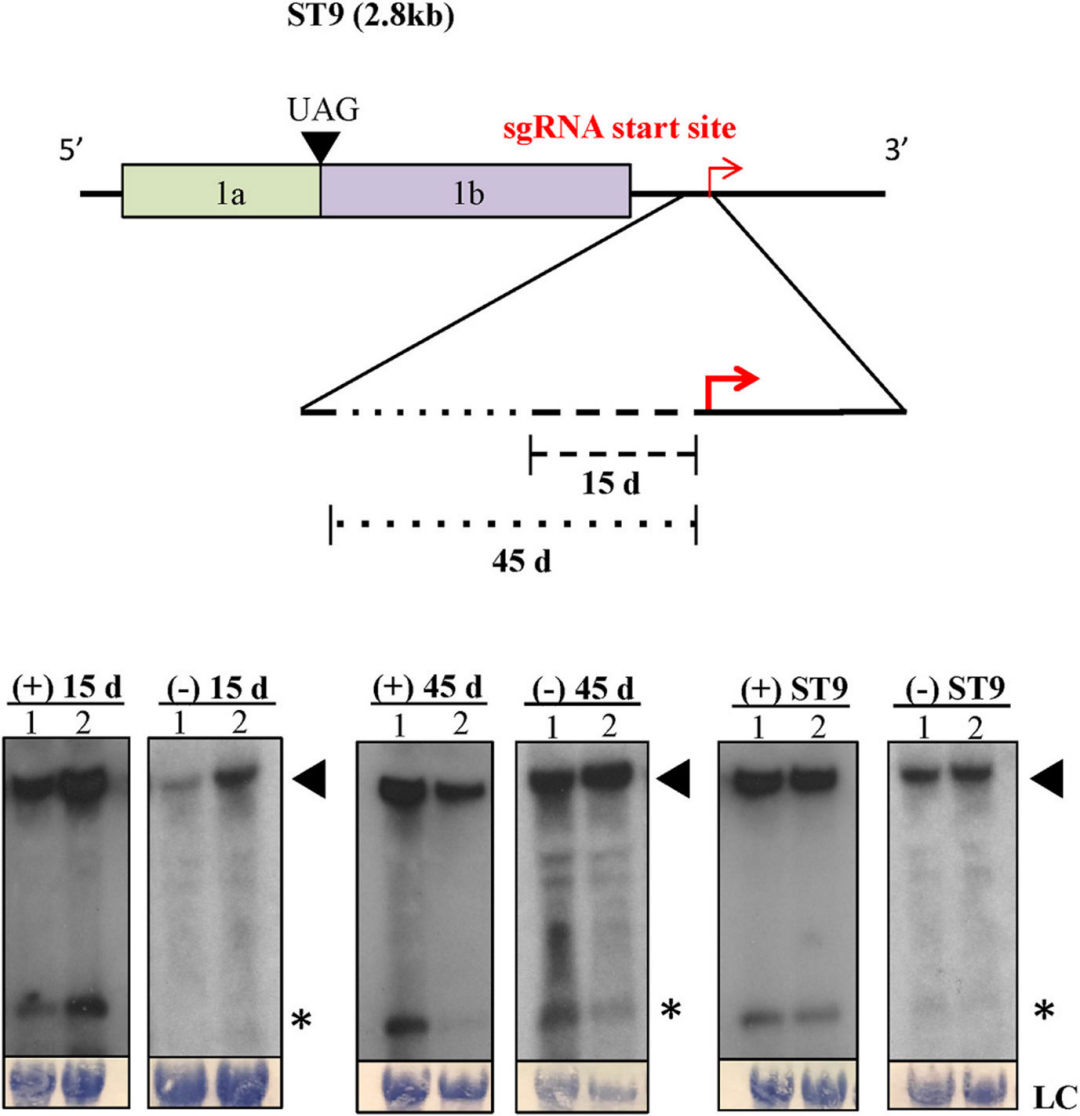
Deletions upstream of the sgRNA start site do not affect sgRNA production or genome replication. The ST9 genome is diagrammed at the top, with a red arrow indicating the 5’ terminus of the sgRNA. A region upstream of the 5’ terminus is shown enlarged below. The overlapping locations of the 15 and 45 nucleotide deletions are shown by dashed and dotted lines respectively. Northern hybridizations for each deletion are shown below, with the wild type ST9 at right. The numbers 1 and 2 indicate samples from two individual plants agroinfiltrated with the construct indicated above the panel. Positive (+) and negative (−) sense RNA is shown, (+) RNAs were exposed for between 2 and 6 h and (−) RNAs were exposed for either 16 h or 4 days. A filled triangle indicates the genomic RNA, an askterisk indicates the sgRNA. Methylene blue stained 28S ribosomal RNA as loading controls are shown at the bottom, LC.

In light of minor changes to the nucleotide sequence surrounding the 5’ end of the sgRNA having no observable effect on ST9, two deletion mutants were created in a further attempt to disable any potential subgenomic promoter driving transcription of the sgRNA. Deletions of 15 and 45 nucleotides respectively were introduced into the genomic RNA immediately upstream of the sgRNA 5’ terminus (Fig. 4). Northern blot hybridization analysis using RNA extracted from plants agroinfiltrated with the deletion mutant constructs showed that neither deletion had any measurable effect on genomic or sgRNA accumulation in either positive or negative RNA polarity (Fig. 4). These results indicate that no sequence or structure in the genomic RNA within 45 nucleotides upstream of the 5’ end of the sgRNA is critical for replication of ST9, or production of its sgRNA. This is an unusual finding for the region upstream of a sgRNA produced by internal initiation of transcription, since the core sequence of most subgenomic promotors lies directly upstream of the 5’ end of the sgRNA. The results presented here strongly suggest that no subgenomic promoter is involved in the production of ST9’s sgRNA and, by extension, that internal initiation of transcription is unlikely to be the mechanism underlying sgRNA generation.

### 3.4. The 3’ UTR of ST9 stalls XRN1 processive decay near the 5’ end of the sgRNA

**Fig. 5.**
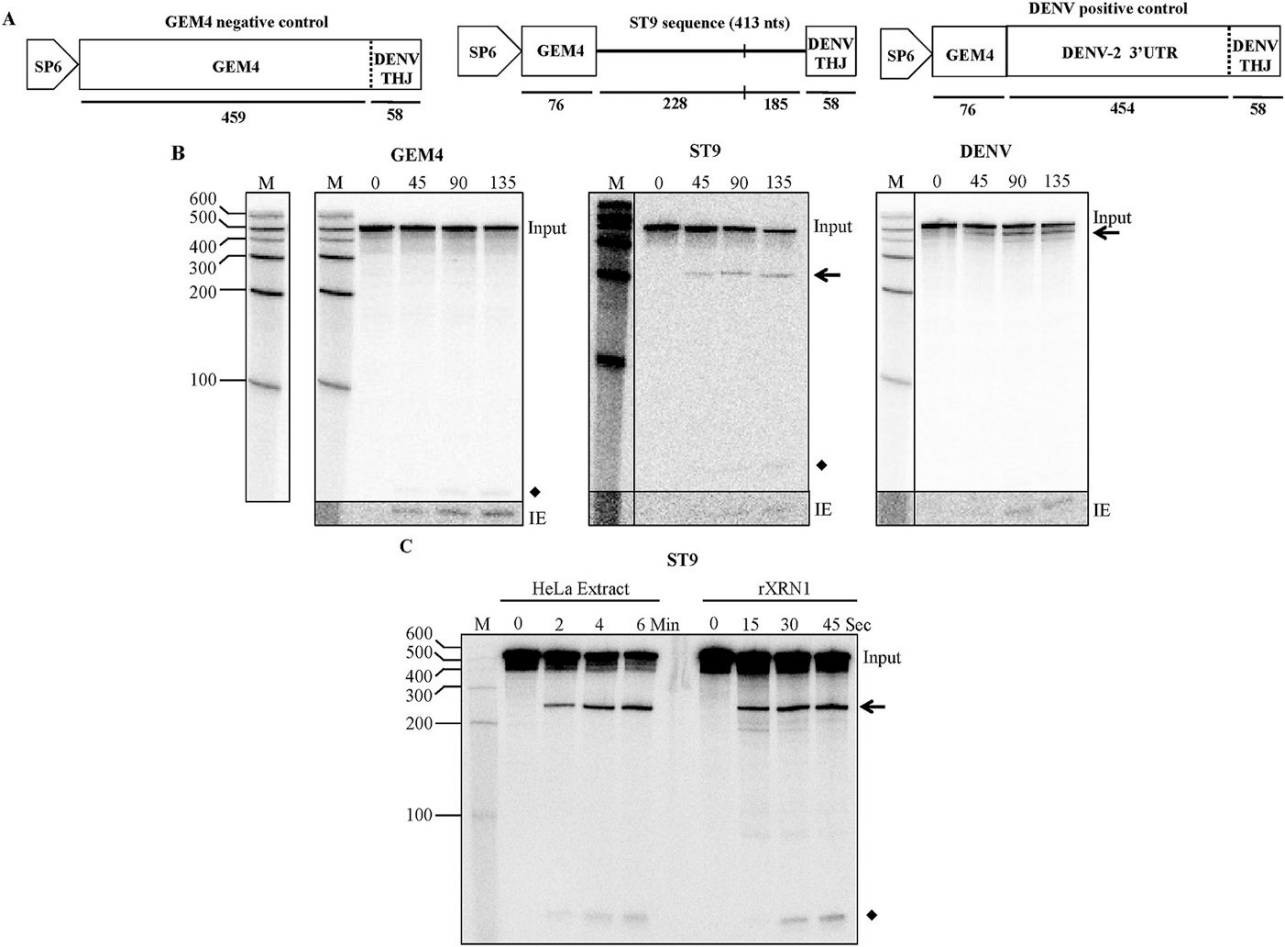
ST9 efficiently resists XRN1 degradation *in vitro.* **A)** Diagrams of the reporter constructs used in all XRN1 decay assays. The GEM4 nonstructured sequence to allow for efficient XRN1 loading is shown boxed; in the GEM4 negative control, all sequence between the SP6 promoter and DENV three helix junction (THJ) sequence is derived from the pGEM4 plasmid. The DENV THJ sequence is indicated by a box; in the positive control a dashed line designates the 5’ end of the DENV THJ, since in this construct all sequence downstream of GEM4 is the wild type DENV-2 3’UTR. In the ST9 construct, 413 nts of ST9 sequence was inserted into the reporter and the 5’ terminus of the sgRNA is indicated with a vertical dash. The nucleotide lengths of each part of the constructs are shown below. **B)** XRN1 decay assays for the GEM negative control, ST9, and the DENV positive control. Samples of the reaction were collected at the times, in seconds, listed at top. The generated decay intermediates are designated with an arrow. The DENV THJ readout intermediate is indicated by a diamond. An increased exposure, IE, of the DENV THJ readout is shown below each panel. The RNA marker, M, loaded in each gel is shown at the left and the sizes of each product in the ladder are given in the panel at the far left. The marker shown next to each gel is the marker loaded in that gel; in the ST9 and DENV panels, the marker was moved to be closer to the sample lanes for ease of size comparison. The GEM and DENV markers are the same, as these reaction products were loaded into the same gel. **C)** XRN1 decay assays using the ST9 construct in reactions with HeLa cell cytoplasmic extract, left, and rXRN1 derived from yeast, right. Samples of the reactions were collected at times indicated at the top. ST9 decay intermediate denoted with an arrow, DENV THJ readout intermediate indicated with a diamond. The RNA marker, M, is shown at left with sizes as indicated.

Since experiments intended to abolish sgRNA production through disabling a potential subgenomic promoter failed to eliminate sgRNA production, the possibility that the major host RNA decay enzyme XRN1 might play a role in accumulation of the ST9 sgRNA as a stable decay intermediate was investigated. We performed *in vitro* decay assays in which the proximal region of the 3’ UTR of ST9 was cloned into a reporter construct, transcribed into 5’ monophosphorylated RNA using radioactively labeled UTP, and incubated with XRN1 (Fig. 5A).

The reporter construct used in these assays contained the ST9 sequence in its 5’ portion while the 3’ end of the reporter construct contained a 58 nucleotide long portion of the dengue virus-2 (DENV) 3’ UTR that forms a three helix junction (THJ) structure that has been shown previously to strongly inhibit and stall XRN1 (Fig. 5A) (Moon et al., 2012, 2015). The DENV-derived structure at the 3’ end serves as an internal control; if nothing preceding it stalls XRN1, a degradation readout product of roughly 58 nucleotides will be present, indicating the exonuclease successfully moved through the body of the RNA but could still be stalled by a known structural element in the 3’ portion of the reporter. RNA reaction products were separated by PAGE and imaged via phosphorimaging.

To assess whether the ST9 3’ UTR was capable of stalling XRN1, 413 nucleotides of ST9 sequence were inserted into the degradation assay reporter construct. This insert encompassed 228 nucleotides upstream and 185 nucleotides downstream of the sgRNA 5’ end. The construct was transcribed *in vitro* into RNA and subjected to XRN1 degradation (Fig. 5). Samples of the reaction were collected over consecutive timepoints to observe the decay process over time. A positive control consisted of a reporter construct containing a larger portion of the wild type DENV-2 3’ UTR which includes the THJ sequence at its 3’ end. As the negative control, additional minimally structured sequence derived from the pGEM4 plasmid (Promega) was inserted upstream of the DENV THJ sequence (Moon et al., 2012) (Fig. 5A).

Over the course of the incubation, a decay intermediate from the ST9 3’ UTR proximal sequence insert was clearly produced (Fig. 5B). Imaging analysis comparing the decay intermediate with the most proximal band of the ladder estimated the decay intermediate to be ~230 nucleotides, placing the XRN1 stall site within roughly 14 nucleotides of the sgRNA start site as determined through 5’ RACE. The assay was similarly performed in HeLa cell cytoplasmic extracts which have been frequently used in XRN1 decay assays due to their high level of enzymatic activity (Boehm et al., 2016; Michalski et al., 2019). The XRN1 degradation assays using HeLa cytoplasmic extracts yielded the same results as the assay with recombinant XRN1 derived from yeast (Fig. 5C). These results clearly demonstrate that a sequence in the 3’ UTR near or at the 5’ end of the sgRNA can efficiently stall XRN1 *in vitro*. This suggests that a similar phenomenon likely takes place *in planta* and that the sgRNA generated by ST9 during infection is produced by exonucleolytic decay enacted on the genomic RNA.

## 4. Discussion

In this work the generation and function of the ST9 sgRNA were investigated and the results strongly suggest that the sgRNA is noncoding and is not generated by internal initiation of transcription. Production of sgRNAs is a common phenomenon among + ssRNA viruses, and several studies have characterized their expression via subgenomic promoters (Grdzelishvili et al., 2000; Koev and Miller, 2000; Miller et al., 1985). Subgenomic promoters range in terms of the size of the core sequence needed for function from about 24 to over 100 nucleotides, and are generally located immediately upstream of the 5’ end of the sgRNA in the genomic context (Miller and Koev, 2000). For example, in the carmovirus turnip crinkle virus (TCV), the minimal sequence required for subgenomic promoter function for the 1.45 kb sgRNA was mapped to a sequence comprising 90 bases upstream of the sgRNA 5’ end and only 6 bases downstream (Wang and Simon, 1997). The Sindbis virus subgenomic promoter was similarly shown to require at least the 18 nucleotides upstream of the sgRNA transcription start site, while only the 5 nucleotides downstream were required to maintain activity (Levis et al., 1990). In the case of ST9, the 45 nucleotides upstream of the 5’ end of the sgRNA were shown to be dispensable for sgRNA production. This result is rare for sgRNA expression mediated by an internal promoter and suggests another mechanism underlies production of the ST9 sgRNA.

This is further supported by the finding that the genome sequence immediately surrounding the 5’ end of the sgRNA is tolerant of substitutions. The seven nucleotide substitution mutants examined in this study had indiscernible effects on the accumulation of the sgRNA. This would be unexpected if the examined sequence was part of a subgenomic promoter since they usually have strict sequence specificity and minor changes have been shown to alter their function. For example, substitution of the first nucleotide of the sgRNA of Striped Jack nervous necrosis virus (family *Nodavirus*) in the genomic context resulted in the ablation of positive sense sgRNA, though the sgRNA accumulated in the negative sense (Iwamoto et al., 2005).

The *in vitro* degradation assays presented here demonstrate that a sequence within the ST9 3’UTR, estimated to include the 5’ end of the sgRNA, is capable of resisting XRN1. Thus, of the two mechanisms for sgRNA generation investigated here, there is strong evidence against internal initiation of transcription, while incomplete degradation of the genomic RNA by 5’-3’ exonucleases is likely to operate in the production of the ST9 sgRNA. It is probable that ST9 resists the homolog of XRN1, XRN4, *in planta,* and that this is responsible for the observed accumulation of the sgRNA.

The work presented here offers an intriguing insight into novel potential roles of ST9 in coinfections. The mutational analysis shown here provides evidence the ST9 sgRNA is likely not a messenger RNA, thus its role in infections is likely carried out as a noncoding RNA. It is also worth noting that other examined tlaRNAs lack ORFs similar to P4, which adds support for the hypothesis that the ORF in the ST9 sgRNA is nonfunctional.

In plants, where coinfections are common, the molecular mechanisms underlying interactions between viruses are receiving more attention. Synergistic interactions are noted in many coinfections with members of the potyvirus family, mediated in part by the role played by the helper component proteinase (HC-Pro) which acts as a potent post transcriptional gene silencing suppressor (Anandalakshmi et al., 1998). A mutualistic interaction is observed between umbraviruses, which lack coat proteins, and the members of the family *Luteoviridae* they associate with. Viruses in the *Luteoviridae* are normally phloem-restricted. However, in coinfections with umbraviruses, they achieve cell-cell movement via utilization of one of two umbravirus movement proteins, while the umbravirus gains encapsidation and thus aphid transmission (Taliansky et al., 2003; Taliansky and Robinson, 2003). Interestingly, one of the two sgRNAs of the umbravirus OPMV is produced through XRN activity and is translated. Mutating the sequence responsible for XRN stalling dramatically reduced the accumulation of genomic and subgenomic RNA, suggesting a critical role for the sgRNA or its encoded protein in infection, facilitated by the host RNA decay system (Ilyas et al., 2021). Another group of coat protein-dependent subviral RNAs related to umbraviruses (ulaRNAs) and only slightly larger than tlaRNAs (2.7–4.6 kb) have recently been described (Liu et al., 2021). Similar to the tlaRNAs, ulaRNAs encode an RdRp for replication yet depend upon coinfection with a viral partner for encapsidation (Liu et al., 2021). Notably, ulaRNAs were shown to be protected against nonsense mediated decay (NMD), a key facet of the host mRNA surveillance and quality control system. NMD in plants is enacted by UPF1 upon detection of lengthy (>200 nts) 3’ UTRs, a feature both of aberrant mRNAs with premature termination codons, and many viruses or subviral RNAs (Kerényi et al., 2008; Kertész et al., 2006). An unspecified element near the 5’ end of the ulaRNA 3’UTR was shown to prevent NMD and represents a candidate molecular mechanism which may influence the interaction between ulaRNAs and coinfection partners (Liu et al., 2021). Collectively, these studies along with the work presented here demonstrate that subviral RNAs can interact with cellular RNA decay factors as a natural part of infection.

Intriguingly, coinfection of ST9 with the polerovirus beet western yellows virus (BWYV) has been shown to produce more severe symptoms and lead to higher accumulation of BWYV RNA than in single infections (11).While the molecular mechanisms underlying the increased symptom severity observed in coinfections of BWYV and tlaRNA ST9 have yet to be elucidated (Falk and Duffus, 1984), it is worth noting the stalling of XRN1 by ST9 RNAs could play a role. Given that the delay of XRN1’s decay activity while stalled on viral RNA structures has been shown to be sufficient to cause dysregulation of gene expression (Moon et al., 2015), an intriguing possibility is that by attracting and stalling XRN1, ST9 genomic RNAs can act as ‘decoys’ to generally impair XRN1’s cytoplasmic function. The benefit yielded to ST9 by thus creating a more favorable environment for viral replication without having to avoid cytoplasmic 5’-3’ exonucleases, for all co-infecting viral partners, may outweigh the cost of producing ‘decoy genomes’ that ultimately will not be used in the infection cycle. While this effect would be derived from the genomic RNA, via the 3’ UTR, a role for the noncoding sgRNA that is produced also needs to be considered. The small RNAs produced upon XRN1 degradation of flavivirus genomes have been shown to be directly responsible for causing cytopathic effects, dysregulation of gene expression, and mortality in animal models (Schuessler et al., 2012; Moon et al., 2012; Michalski et al., 2019). The resistance of ulaRNAs against NMD and tlaRNAs against XRN1 offers an intriguing view into how subviral noncoding RNAs could significantly impact the viral partners they depend upon in coinfections through subversion of the host RNA decay system and perhaps other aspects of the RNA biology of the cell. The results presented here lay clear foundations for future research to address these enticing questions about the ways in which viruses and subviral RNAs can interact with one another upon coinfection.

## Supplementary Material

Campbell et al, Supplementary Material

## Figures and Tables

**Table 1:** tlaRNA sgRNA start site determination via 5’ RACE.

tlaRNA	Subclade	sgRNA start site	No. clones with indicated start site/total sequenced	sgRNA length
ST9	II	A2378	8/10	467 nts
Alpha	I	C2363	16/22	471 nts
Gamma	I	C2365	5/7	471 nts
Sigma	I	C2358	12/17	517 nts

## References

[R1] Akiyama BM, Laurence HM, Massey AR, Costantino DA, Xie X, Yang Y, Shi P-Y, Nix JC, Beckham JD, Kieft JS, 2016. Zika virus produces noncoding RNAs using a multi-pseudoknot structure that confounds a cellular exonuclease. Science (80-. ) 354, 1148. 10.1126/science.aah3963. PMC5476369 27934765

[R2] Anandalakshmi R, Pruss GJ, Ge X, Marathe R, Mallory AC, Smith TH, Vance VB, 1998. A viral suppressor of gene silencing in plants. Proc. Natl. Acad. Sci. Unit. States Am 95, 13079–13084. 10.1073/pnas.95.22.13079 PMC23715 9789044

[R3] Boehm V, Gerbracht JV, Marx MC, Gehring NH, 2016. Interrogating the degradation pathways of unstable mRNAs with XRN1-resistant sequences. Nat. Commun 7 10.1038/ncomms13691. PMC5150221 27917860

[R4] Campbell AJ; Erickson A; Pellerin E; Salem N; Mo X; Falk BW; Ferriol I Phylogenetic classification of a group of self-replicating RNAs that are common in co-infections with poleroviruses. Virus Res.. 2020, 197831, doi:10.1016/j.virusres.2019.197831. 31790776

[R5] Chapman EG, Costantino DA, Rabe JL, Moon SL, Wilusz J, Nix JC, Kieft JS, 2014. The structural basis of pathogenic subgenomic flavivirus RNA (sfRNA) production. Science (80-. ) 344, 307–310. 10.1126/science.1250897. PMC4163914 24744377

[R6] Charley PA, Wilusz CJ, Wilusz J, 2018. Identification of phlebovirus and arenavirus RNA sequences that stall and repress the exoribonuclease XRN1. J. Biol. Chem 293, 285–295. 10.1074/jbc.M117.805796. 29118186 PMC5766927

[R7] Chin L-S, Foster JL, Falk BW, 1993. The beet Western yellows virus ST9-associated RNA shares structural and nucleotide sequence homology with carmo-like viruses. Virology 192, 473–482. 10.1006/VIRO.1993.1063. 8421895

[R8] Choi IR, Andrew White K, 2002. An RNA activator of subgenomic mRNA1 transcription in tomato bushy stunt virus. J. Biol. Chem 277, 3760–3766. 10.1074/jbc.M109067200. 11714712

[R9] Dickson AM, Wilusz J, 2011. Strategies for viral RNA stability: live long and prosper. Trends Genet. 27, 286–293. 21640425 10.1016/j.tig.2011.04.003 PMC3123725

[R10] Dilweg IW, Gultyaev AP, Olsthoorn RC, 2019. Structural features of an Xrn1-resistant plant virus RNA. RNA Biol. 16, 838–845. 10.1080/15476286.2019.1592070. 30951405 PMC6546385

[R11] Eckerle LD, Ball LA, 2002. Replication of the RNA segments of a bipartite viral genome is coordinated by a transactivating subgenomic RNA. Virology 296, 165–176. 10.1006/viro.2002.1377. 12036328

[R12] Falk BW, Duffus JE, 1984. Identification of small single-and double-stranded RNAs associated with severe symptoms in Beet western yellows virus-infected Capsella bursa-pastoris. Phytopathology 1224–1229.

[R13] Grdzelishvili VZ, Chapman SN, Dawson WO, Lewandowski DJ, 2000. Mapping of the Tobacco mosaic virus movement protein and coat protein subgenomic RNA promoters in vivo. Virology 275, 177–192. 10.1006/viro.2000.0511. 11017798

[R14] Gunawardene CD, Newburn LR, Andrew White KA, 2019. 212-nt long RNA structure in the Tobacco necrosis virus-D RNA genome is resistant to Xrn degradation. Nucleic Acids Res. 47, 9329–9342. 10.1093/nar/gkz668. 31392982 PMC6755097

[R15] Gupta P, Rangan L, Ramesh TV, Gupta M, 2016. Comparative analysis of contextual bias around the translation initiation sites in plant genomes. J. Theor. Biol 404, 303–311. 10.1016/j.jtbi.2016.06.015. 27316311

[R16] Hernández G, Osnaya VG, Pérez-Martínez X, 2019. Conservation and variability of the AUG initiation codon context in Eukaryotes. Trends Biochem. Sci 44, 1009–1021. 31353284 10.1016/j.tibs.2019.07.001

[R17] Ilyas M, Du Z, Simon AE, 2021. Opium poppy mosaic virus has an Xrn-resistant, translated subgenomic RNA and a BTE *3’* CITE. J. Virol 95. 10.1128/jvi.02109-20. PMC8104115 33597210

[R18] Iwamoto T, Mise K, Takeda A, Okinaka Y, Mori KI, Arimoto M, Okuno T, Nakai T, 2005. Characterization of striped jack nervous necrosis virus subgenomic RNA3 and biological activities of its encoded protein B2. J. Gen. Virol 86, 2807–2816. 10.1099/vir.0.80902-0. 16186236

[R19] Johnston JC, Rochon DM, 1995. Deletion analysis of the promoter for the cucumber necrosis virus 0.9-kb subgenomic RNA. Virology 214, 100–109. 8525604 10.1006/viro.1995.9950

[R20] Kerényi Z, Mérai Z, Hiripi L, Benkovics A, Gyula P, Lacomme C, Barta E, Nagy F, Silhavy D, 2008. Inter-kingdom conservation of mechanism of nonsense-mediated mRNA decay. EMBO J. 27, 1585–1595. 10.1038/emboj.2008.88. 18451801 PMC2426726

[R21] Kertész S, Kerényi Z, Mérai Z, Bartos I, Pálfy T, Barta E, Silhavy D, 2006. Both introns and long 3′-UTRs operate as cis-acting elements to trigger nonsense-mediated decay in plants. Nucleic Acids Res. 34, 6147–6157. 10.1093/nar/gkl737. 17088291 PMC1693880

[R22] Kieft JS, Rabe JL, Chapman EG, 2015. New hypotheses derived from the structure of a flaviviral Xrn1-resistant RNA: conservation, folding, and host adaptation. RNA Biol. 12, 1117–1169. 10.1080/15476286.2015.1094599. PMC4829329 26399159

[R23] Koev G, Miller WA, 2000. A Positive-Strand RNA Virus with Three Very Different Subgenomic RNA Promoters, 74. 10.1128/jvi.74.13.5988-5996.2000 PMC112095 10846080

[R24] Levis R, Schlesinger S, Huang HV, 1990. Promoter for Sindbis virus RNA-dependent subgenomic RNA transcription. J. Virol 64, 1726–1733. 2319651 10.1128/jvi.64.4.1726-1733.1990 PMC249310

[R25] Lindbo JA, 2007. TRBO: A high-efficiency tobacco mosaic virus RNA-based overexpression vector. Plant Physiol. 145, 1232–1240. 10.1104/pp.107.106377. 17720752 PMC2151719

[R26] Liu J, Carino E, Bera S, Gao F, May JP, Simon AE, 2021. Structural analysis and whole genome mapping of a new type of plant virus subviral RNA: umbravirus-like associated RNAs. Viruses 13. 10.3390/v13040646. PMC8068935 33918656

[R27] Matsumura EE, Coletta-Filho HD, Machado MA, Nouri S, Falk BW, 2019. Rescue of Citrus sudden death-associated virus in Nicotiana benthamiana plants from cloned cDNA: insights into mechanisms of expression of the three capsid proteins. Mol. Plant Pathol 20, 611–625. 10.1111/mpp.12780. 30575252 PMC6637869

[R28] Michalski D, Gustavo Ontiveros J, Russo J, Charley PA, Anderson JR, Heck AM, Geiss BJ, Wilusz J, 2019. Zika virus noncoding sfRNAs sequester multiple host-derived RNA-binding proteins and modulate mRNA decay and splicing during infection. J. Biol. Chem 294, 16282–16296. 10.1074/jbc.RA119.009129. 31519749 PMC6827284

[R29] Miller WA, Koev G, 2000. Synthesis of subgenomic RNAs by positive-strand RNA viruses. Virology 273, 1–8. 10891401 10.1006/viro.2000.0421

[R30] Miller WA, White KA, 2006. Long-distance RNA-RNA interactions in plant virus gene expression and replication. Annu. Rev. Phytopathol 44, 447–467. 10.1146/annurev.phyto.44.070505.143353. 16704356 PMC1894749

[R31] Miller WA, Dreher TW, Hall TC, 1985. Synthesis of brome mosaic virus subgenomic RNA in vitro by internal initiation on (−)-sense genomic RNA. Nature 313. 10.1038/313068a0 3838107

[R32] Miller AW, Liu S, Beckett R, 2002. Barley yellow dwarf virus: Luteoviridae or Tombusviridae? Mol. Plant Pathol 3, 177–183. 20569325 10.1046/j.1364-3703.2002.00112.x

[R33] Moon SL, Anderson JR, Kumagai Y, Wilusz CJ, Akira S, Khromykh AA, Wilusz J, 2012. A noncoding RNA produced by arthropod-borne flaviviruses inhibits the cellular exoribonuclease XRN1 and alters host mRNA stability. RNA 18, 2029–2040. 10.1261/rna.034330.112. 23006624 PMC3479393

[R34] Moon SL, Blackinton JG, Anderson JR, Dozier MK, Dodd BJT, Keene JD, Wilusz CJ, Bradrick SS, Wilusz J, 2015. XRN1 stalling in the 5’ UTR of hepatitis C virus and bovine viral diarrhea virus is associated with dysregulated host mRNA stability. PLoS Pathog. 11, 1–21. 10.1371/journal.ppat.1004708. PMC4352041 25747802

[R35] Moreno AB, López-Moya JJ, 2020. When viruses play team sports: mixed infections in plants. Phytopathology 110, 29–48. 10.1094/PHYTO-07-19-0250-FI. 31544593

[R36] Na H, Fabian MR, White KA, 2006. Conformational organization of the 3′ untranslated region in the tomato bushy stunt virus genome. RNA 12, 2199–2210. 10.1261/rna.238606. 17077273 PMC1664717

[R37] Passmore BK, Sanger M, Chin LS, Falk BW, Bruening G, 1993. Beet western yellows virus-associated RNA: an independently replicating RNA that stimulates virus accumulation. Proc. Natl. Acad. Sci. U. S. A 90, 10168–10172. 8234272 10.1073/pnas.90.21.10168 PMC47735

[R38] Peng J, Bu S, Yin Y, Hua M, Zhao K, Lu Y, Zheng H, Wan Q, Zhang S, Chen H, , 2021. Biological and genetic characterization of pod pepper vein yellows virus-associated RNA from capsicum frutescens in Wenshan, China. Front. Microbiol 12. 10.3389/fmicb.2021.662352. PMC8083956 33936020

[R39] Pijlman GP, Funk A, Kondratieva N, Leung J, Torres S, van der Aa L, Liu WJ, Palmenberg AC, Shi PY, Hall RA, , 2008. A highly structured, nuclease-resistant, noncoding RNA produced by flaviviruses is required for pathogenicity. Cell Host Microbe 4, 579–591. 10.1016/j.chom.2008.10.007. 19064258

[R40] Pompon J, Manuel M, Ng GK, Wong B, Shan C, Manokaran G, Soto-Acosta R, Bradrick SS, Ooi EE, Missé D, , 2017. Dengue subgenomic flaviviral RNA disrupts immunity in mosquito salivary glands to increase virus transmission. PLoS Pathog. 13 10.1371/journal.ppat.1006535. PMC5555716 28753642

[R41] Qiao W, Falk BW, 2018. Efficient protein expression and virus-induced gene silencing in plants using a crinivirus-derived vector. Viruses 10. 10.3390/v10050216. PMC5977209 29695039

[R42] Qiao W, Helpio EL, Falk BW, 2018. Two crinivirus-conserved small proteins, P5 and P9, are indispensable for efficient Lettuce infectious yellows virus infectivity in plants. Viruses 10. 10.3390/v10090459. PMC6163742 30154314

[R43] Sanger M, Passmore B, Falk BW, Bruening G, Ding B, Lucas WJ, 1994. Symptom severity of beet western yellows virus strain ST9 is conferred by the ST9-associated RNA and is not associated with virus release from the phloem. Virology 200, 48–55. 10.1006/viro.1994.1161. 8128637

[R44] Schuessler A, Funk A, Lazear HM, Cooper DA, Torres S, Daffis S, Jha BK, Kumagai Y, Takeuchi O, Hertzog P, , 2012. West nile virus noncoding subgenomic RNA contributes to viral evasion of the type I Interferon-mediated antiviral response. J. Virol 86, 5708–5718. 10.1128/jvi.00207-12. 22379089 PMC3347305

[R45] Shen R, Miller WA, 2004. Subgenomic RNA as a riboregulator: negative regulation of RNA replication by Barley yellow dwarf virus subgenomic RNA 2. Virology 327, 196–205. 10.1016/j.virol.2004.06.025. 15351207

[R46] Siegel RW, Adkins S, Cheng Kao C, 1997. Sequence-specific recognition of a subgenomic RNA promoter by a viral RNA polymerase. Biochemistry 94, 11238–11243. 10.1073/pnas.94.21.11238 PMC23427 9326593

[R47] Slonchak A, Khromykh AA, 2018. Subgenomic flaviviral RNAs: what do we know after the first decade of research. Antivir. Res 159, 13–25. 30217649 10.1016/j.antiviral.2018.09.006

[R48] Steckelberg A-L, Akiyama BM, Costantino DA, Sit TL, Nix JC, Kieft JS, 2018a. A folded viral noncoding RNA blocks host cell exoribonucleases through a conformationally dynamic RNA structure. Proc. Natl. Acad. Sci 115, 6404–6409. 10.1073/pnas.1802429115. 29866852 PMC6016793

[R49] Steckelberg AL, Vicens Q, Kieft JS, 2018b. Exoribonuclease-resistant RNAs exist within both coding and noncoding subgenomic RNAs. mBio 9. 10.1128/mBio.02461-18. PMC6299227 30563900

[R50] Sztuba-Solińska J, Stollar V, Bujarski JJ, 2011. Subgenomic messenger RNAs: mastering regulation of (+)-strand RNA virus life cycle. Virology. 10.1016/j.virol.2011.02.007 PMC7111999 21377709

[R51] Taliansky ME, Robinson DJ, 2003. Molecular biology of umbraviruses: phantom warriors. J. Gen. Virol 84, 1951–1960. 12867625 10.1099/vir.0.19219-0

[R52] Taliansky M, Roberts IM, Kalinina N, Ryabov EV, Raj SK, Robinson DJ, Oparka KJ, 2003. An umbraviral protein, involved in long-distance RNA movement, binds viral RNA and forms unique, protective ribonucleoprotein complexes. J. Virol 77, 3031–3040. 10.1128/jvi.77.5.3031-3040.2003. 12584328 PMC149777

[R53] Tycowski KT, Guo YE, Lee N, Moss WN, Vallery TK, Xie M, Steitz JA, 2015. Viral noncoding RNAs: more surprises. Genes Dev. 29, 567–584. 25792595 10.1101/gad.259077.115 PMC4378190

[R54] Van der Vossen E, Notenboom T, Bol J, 1995. Characterization of sequences controlling the synthesis of alfalfa mosaic virus subgenomic RNA in Vivo. Virology 212. 10.1006/viro.1995.1524 7571436

[R55] Wang J, Simon AE, 1997. Analysis of the two subgenomic RNA promoters for turnip crinkle virus in vivo and in vitro. Virology 232, 174–186. 9185601 10.1006/viro.1997.8550

[R56] Wang J, Turina M, Medina V, Falk BW, 2009. Synergistic interaction between the Potyvirus, Turnip mosaic virus and the Crinivirus, Lettuce infectious yellows virus in plants and protoplasts. Virus Res. 144, 163–170. 10.1016/j.virusres.2009.04.017. 19409943

[R57] White KA, 2002. The premature termination model: a possible third mechanism for subgenomic mRNA transcription in (+)-strand RNA viruses. Virology 304, 147–154. 12504556 10.1006/viro.2002.1732

