# Supplementary material for: A plant-infecting subviral RNA associated with poleroviruses produces a subgenomic RNA which resists exonuclease XRN1 in vitro
Source: Virology. Author manuscript; Available in PMC 2023 Jan 11. (PMC9832584; doi:10.1016/j.virol.2021.11.002)
Supplement: Campbell et al, Supplementary Material [file NIHMS1858663-supplement-Campbell_et_al__Supplementary_Material.pdf]

Supplement: A plant-infecting subviral RNA associated with poleroviruses produces a subgenomic RNA which resists host exonuclease XRN1 *in vitro*

Supplementary Table 1. Primers used in all experiments

Objective	Primer name	Sequence (5'-3')
**ST9 sgRNA Northern Mapping**	ST9 T7 p1 F	ACCGTAATACGACTCACTATAGGGAGAATGTCTAGCCATCTGTTGAGC
	ST9 p1 R	GATTGAGTTCTGCTCGGTCTC
	ST9 T7 p2 F	AGGCTAATACGACTCACTATAGGGAGACATAAGAGTTCTCCGAGCCG
	ST9 p2 R	GGCAAGTATCGGTCCACC
	ST9 T7 p3 F	ACCGTAATACGACTCACTATAGGGAGACATGGCGGAGCCTATCAGTAATTG
	ST9 p3 R	TCATGTAGTTGCCCCTTCTG
	ST9 T7 p4 F	AGGCTAATACGACTCACTATAGGGAGACGTGGCGATCTGTCTTGAC
	ST9 p4 R	CTCTACCTTGAGCAGGAATCCG
	ST9 T7 p5 F	AGGCTAATACGACTCACTATAGGGAGACAGCTCACTCCTGATGTATG
	ST9 p5 R	TTAAACATCACCTTCACACACTGG
	ST9 T7 p6 F	ACCGTAATACGACTCACTATAGGGAGATGGTAGATCGCACGCGGC
	ST9 p6 R	GGGCGGTTGACCTGGTATTG
**5' RACE**	Anchor Adaptor Primer	GGCCACGCGTCGACTAGTACTTTTTTTTTTTTTTTTT
	Abridged adapter primer	GGCCACGCGTCGACTAGTAC
	ST9 GSP 2	CCAAGTGGGTGTTAGTCACG
	ST9 GSP 3	CGATGTCCACCCAAGAGTTCGAGTG
	Alpha/Gamma GSP 3	GAATGTTGGAGCGTGGGCAAGC
	Alpha GSP 2R	CCATCAACTATTCCATAGACC
	Gamma GSP 2R	TCCATAGATCCCAAGCATGGC
	Sigma GSP 2R	GGCTCGGAAGCTAATTAAGACC
	Sigma GSP 3R	CGGAAGAGCCGCTTTACTCTAC
**P4 KO mutant**	Met-Ala F	TCGCACGCGGCATTAAACCTGCTGTGCATCCGGTCAGAATC
	Met-Ala R	CAGGTTTAATGCCGCGTGCGATCTACCGCATGTCGTGTGG
**sgRNA start site substitutions**	1nt A-G F	GAGTCATAACAACGAGTACCCTCCAAGCCCTACAG
	1nt A-G R	GAGGGTACTCGTTGTTATGACTCTATCCTAACTAG
	1nt A-T F	CATAACAACTAGTACCCTCCAAGCCCTACAGG
	1nt A-T R	CTTGGAGGGTACTAGTTGTTATGACTCTATCCTAACTAG
	1nt C-T F	CATAACAATAAGTACCCTCCAAGCCCTACAGG
	1nt C-T R	CTTGGAGGGTACTTATTGTTATGACTCTATCC
	2nt AG-TT F	CATAACAACTATTACCCTCCAAGCCCTACAGG
	2nt AG-TT R	GCTTGGAGGGTAATAGTTGTTATGACTCTATCC
	2nt CC-TT F	CATAATAATAAGTACCCTCCAAGCCCTACAGGTTGGAAGAG
	2nt CC-TT R	GCTTGGAGGGTACTTATTATTATGACTCTATCCTAACTAG
	3nt CAG-TTT F	CAATTATTACCCTCCAAGCCCTACAGGTTGGAAG
	3nt CAG-TTT R	GGCTTGGAGGGTAATAATTGTTATGACTCTATCC
	3nt CCA-TTG F	GTCATAATAATGAGTACCCTCCAAGCCCTACAGGTTG
	3nt CCA-TTG R	GGTACTCATTATTATGACTCTATCCTAACTAG
**Sgpromoter deletions**	ST9 SgP del F	CAACAAGTACCCTCCAAGCCCTACAGGTTGGAAGAG
	15nt del R	AGGGCTTGGAGGGTACTTGTTGAACTAGAAATATTATACTAATGGTGAC
	45nt del R	AGGGCTTGGAGGGTACTTGTTGTCATTCTTCCTAGTGAGGTGGTCCCTTGATCC
**Northern blot probes**	ST9 T7 pos R	ACCGTAATACGACTCACTATAGGGAGAGGGCGGTTGACCTGGTATTG
	ST9 pos F	CACACGACATATGGTAGATCGCAC
	ST9 T7 neg F	ACCGTAATACGACTCACTATAGGGAGATGGTAGATCGCACGCGGC
	ST9 neg R	GGGCGGTTGACCTGGTATTG

Supplementary Table 2. Sequences of XRN1 decay assay construct templates

ST9 reporter contstruct	**CATACGATTTAGGTGACACTATA**gaatacacggaattcgagctcggtacccggggatcctctagagtcgacctgc<br>aggcatgcaagcttccggtctcccATAGAGTAGAATTTGTACATAGTTTTCTAGGTGACTATCCAG<br>TGTGTGAAGGTGATGTTTAATACACTACATGCGTCTTGGCTTACGTGAGCCAATGCC<br>GCGACCTCTGCCCTTAGGAGTTCAGAAAAGCTGATACCAAGTCATTGGATCAAGGG<br>ACCACCTCACTAGGAAGAATGAAACTGTCACCATTAGTATAATATTTCTAGTTAGGA<br>TAGAGTCATAACAAC**A**AGTACCCTCCAAGCCCTACAGGTTGGAAGAGGGGGCTATC<br>AGTCCTGTAGGCAGACTCGCGTCTCCTGGCGCCACCCCACTCGAACTCTTGGGTGGA<br>CATCGCCAGTCTCGTATACACCGGGGAGAGAGACGACACCCAACTAGGGGGGCCAA<br>TGGCCTAGTGGTTACTGGAAGCCTAATCCAGAGAAGAAGTCAGGCCCCAAAGCCAC<br>GGTTTGAGCAAACCGTGCTGCCTGTAGCTCCGTC
GEM4 negative control reporter construct	**CATACGATTTAGGTGACACTATA**gaatacacggaattcgagctcggtacccggggatcctctagagtcgacctgc<br>aggcatgcaagcttccggtctcccTATAGTGAGTCGTATTAATTTCGATAAGCCAGCTGCATTAAT<br>GAATCGGCCAACGCGCGGGGAGAGGCGGTTTGCGTATTGGGCGCTCTTCCGCTTCCT<br>CGCTCACTGACTCGCTGCGCTCGGTCGTTCGGCTGCGGCGAGCGGTATCAGCTCACT<br>CAAAGGCGGTAATACGGTTATCCACAGAATCAGGGGATAACGCAGGAAAGAACATG<br>TGAGCAAAAGGCCAGCAAAAGGCCAGGAACCGTAAAAAGGCCGCGTTGCTGGCGTT<br>TTTCCATAGGCTCCGCCCCCCTGACGAGCATCACAAAAATCGACGCTCAAGTCAGAG<br>GTGGCGAAACCCGACAGGACTATAAAGATACCAGGCGTTTCCCCCTGGAAGCTCCCT<br>CGAAGAAGTCAGGCCCCAAAGCCACGGTTTGAGCAAACCGTGCTGCCTGTAGCTCC<br>GTC
DENV positive control reporter construct	**CATACGATTTAGGTGACACTATA**gaatacacggaattcgagctcggtacccggggatcctctagagtcgacctgc<br>aggcatgcaagcttccggtctcccTAGAAGGCAAAACTAACATGAAACAAGGCTAGAAGTCAGGT<br>CGGATTAAGCCATAGTACGGAAAAAACTATGCTACCTGTGAGCCCCGTCCAAGGAC<br>GTTAAAAGAAGTCAGGCCATTACAAATGCCATAGCTTGAGTAAACTGTGCAGCCTGT<br>AGCTCCACCTGAGAAGGTGTAAAAAATCTGGGAGGCCACAAACCATGGAAGCTGTA<br>CGCATGGCGTAGTGGACTAGCGGTTAGAGGAGACCCCTCCCTTACAAATCGCAGCA<br>ACAATGGGGGCCCAAGGTGAGATGAAGCTGTAGTCTCACTGGAAGGACTAGAGGTT<br>AGAGGAGACCCCCCCAAAACAAAAAACAGCATATTGACGCTGGGAAAGACCAGAG<br>ATCCTGCTGTCTCCTCAGCATCATTCCAGGCACAGAACGCCAGAAAATGGAATGGTG<br>CTGTTGAATCAACAGGTTCTGAAGAAGTCAGGCCCCAAAGCCACGGTTTGAGCAAA<br>CCGTGCTGCCTGTAGCTCCGTC

The DNA sequences of the constructs used in the XRN1 decay assays are given at right. The SP6 promoter sequence is indicated by bold, red type. The DENV three helix junction (THJ) sequence that yields the readout intermediate is shown in blue, underlined type. The pGEM4 nonstructured sequence that serves as an optimal substrate for XRN1 is shown in lowercase font, except for the GEM4 negative control construct, where the entire sequence between the SP6 promoter and the DENV THJ is derived from the pGEM4 commercially available plasmid. The 5′ terminal nucleotide of the ST9 sgRNA as determined by 5′ RACE is indicated in bold, underlined type.
